# Better COVID-19 Intensive Care Unit survival in females, independent of age, disease severity, comorbidities, and treatment

**DOI:** 10.1038/s41598-021-04531-x

**Published:** 2022-01-14

**Authors:** Daniek A. M. Meijs, Bas C. T. van Bussel, Björn Stessel, Jannet Mehagnoul-Schipper, Anisa Hana, Clarissa I. E. Scheeren, Sanne A. E. Peters, Walther N. K. A. van Mook, Iwan C. C. van der Horst, Gernot Marx, Dieter Mesotten, Chahinda Ghossein-Doha, CoDaP investigators, Nanon F. L. Heijnen, Johannes Bickenbach, Meta C. E. van der Woude, Anne Raafs, Sander M. J. van Kuijk, Luc J. M. Smits, Emma B. N. J. Janssen, Noёlla Pierlet, Ben Goethuys, Jonas Bruggen, Gilles Vermeiren, Hendrik Vervloessem, Mark M. G. Mulder, Marcel Koelmann, Julia L. M. Bels, Laura Bormans-Russell, Micheline C. D. M. Florack, Willem Boer, Margot Vander Laenen

**Affiliations:** 1 Department of Intensive Care Medicine, Maastricht University Medical Center + (Maastricht UMC+), P. Debyelaan 25, 6229 HX Maastricht, the Netherlands; 2 Department of Intensive Care Medicine, Laurentius Ziekenhuis, Roermond, the Netherlands; 3 Present Address: Care and Public Health Research Institute (CAPHRI), Maastricht University, Maastricht, the Netherlands; 4 Department of Intensive Care Medicine, Jessa Hospital, Hasselt, Belgium; 5 Department of Intensive Care Medicine, VieCuri Medisch Centrum, Venlo, the Netherlands; 6 Department of Intensive Care Medicine, Zuyderland Medisch Centrum, Heerlen/Sittard, the Netherlands; 7 Julius Center for Health Sciences and Primary Care, University Medical Center Utrecht, Utrecht, the Netherlands; 8 The George Institute for Global Health, Imperial College London, London, United Kingdom; 9 The George Institute for Global Health, University of New South Wales, Sydney, Australia; 10 Maastricht UMC+ Academy for Postgraduate Medical Education, Maastricht, the Netherlands; 11 Cardiovascular Research Institute Maastricht (CARIM), Maastricht, the Netherlands; 12 Department of Intensive Care Medicine, University Hospital Rheinisch Westfälische Hochschule (RWTH) Aachen, Aachen, Germany; 13 Department of Intensive Care Medicine, Ziekenhuis Oost-Limburg, Genk, Belgium; 14 Faculty of Medicine and Life Sciences, UHasselt, Diepenbeek, Belgium; 15 Department of Cardiology, Maastricht UMC+, Maastricht, the Netherlands; 16 School for Oncology and Developmental Biology, Maastricht UMC+, Maastricht, the Netherlands; 17 Present Address: Department of Clinical Epidemiology and Medical Technology Assessment, Maastricht UMC+, Maastricht, the Netherlands

**Keywords:** Diseases, Medical research, Pathogenesis, Risk factors

## Abstract

Although male Severe Acute Respiratory Syndrome Coronavirus 2 (SARS-CoV-2) patients have higher Intensive Care Unit (ICU) admission rates and a worse disease course, a comprehensive analysis of female and male ICU survival and underlying factors such as comorbidities, risk factors, and/or anti-infection/inflammatory therapy administration is currently lacking. Therefore, we investigated the association between sex and ICU survival, adjusting for these and other variables. In this multicenter observational cohort study, all patients with SARS-CoV-2 pneumonia admitted to seven ICUs in one region across Belgium, The Netherlands, and Germany, and requiring vital organ support during the first pandemic wave were included. With a random intercept for a center, mixed-effects logistic regression was used to investigate the association between sex and ICU survival. Models were adjusted for age, Acute Physiology and Chronic Health Evaluation II (APACHE II) score, comorbidities, and anti-infection/inflammatory therapy. Interaction terms were added to investigate effect modifications by sex with country and sex with obesity. A total of 551 patients (29% were females) were included. Mean age was 65.4 ± 11.2 years. Females were more often obese and smoked less frequently than males (p-value 0.001 and 0.042, respectively). APACHE II scores of females and males were comparable. Overall, ICU mortality was 12% lower in females than males (27% vs 39% respectively, p-value < 0.01) with an odds ratio (OR) of 0.62 (95%CI 0.39–0.96, p-value 0.032) after adjustment for age and APACHE II score, 0.63 (95%CI 0.40–0.99, p-value 0.044) after additional adjustment for comorbidities, and 0.63 (95%CI 0.39–0.99, p-value 0.047) after adjustment for anti-infection/inflammatory therapy. No effect modifications by sex with country and sex with obesity were found (p-values for interaction > 0.23 and 0.84, respectively). ICU survival in female SARS-CoV-2 patients was higher than in male patients, independent of age, disease severity, smoking, obesity, comorbidities, anti-infection/inflammatory therapy, and country. Sex-specific biological mechanisms may play a role, emphasizing the need to address diversity, such as more sex-specific prediction, prognostic, and therapeutic approach strategies.

## Introduction

In early 2020, a novel β-coronavirus causing Severe Acute Respiratory Syndrome Coronavirus 2 (SARS-CoV-2) rapidly spread worldwide, resulting in a pandemic with global impact with an important proportion of the hospitalized patients requiring supportive treatment in the Intensive Care Unit (ICU)^1,2^. By now, risk factors such as age, smoking, cardiovascular and pulmonary comorbidities have been ascertained to influence fatality rates in hospitalized patients strongly^3^. Although Coronavirus Disease 2019 (COVID-19) infects both sexes at similar incidence, case-fatality rates are lower for females (7%) than males (10%), resulting in a marked male-to-female case fatality ratio ranging from 1.6 to 2.8, according to the Global Health 50/50 data tracker^4,5^. Furthermore, a systematic review and meta-analysis of more than 3 million cases demonstrated that male sex was associated with higher odds ratios of requiring ICU admission and overall mortality^6^. On the other hand, an Italian multicenter study revealed higher ICU admission rates for males than females^7,8^. Furthermore, multi-organ failure in the ICU has appeared to develop more favorable in surviving females than males^9^. Previous research confirmed the findings that male patients predominate in the ICU and receive more supportive treatment than females^10–12^.

Sex contributed to disparities in vulnerability, incidence, and case-fatality rates of various diseases in the past^4,13^.

It is assumed that sex differences affect viral susceptibility, response to the virus, disease course, and (side-) effects of initiated therapy, emphasizing why sex aspects and sex-specific data analyses should be implemented in clinical studies^14,15^. We recently demonstrated that current clinical trials on pharmacological therapies for COVID-19 rarely report sex-stratified analyses^16^, which illustrates that sex differences are infrequently taken into account.

Although some studies have depicted higher mortality rates in male patients^6,17–19^, these studies have been performed in heterogeneous populations with clinical diversity, such as a broad range of disease courses, ranging from mild symptoms to respiratory insufficiency requiring ICU admission^20^. More importantly, other baseline risk factors known to be associated with poor outcome, such as age, comorbidities, disease severity, and therapy, are often not taken into account^21^. To conclude, a comprehensive analysis of female and male survival of COVID-19 in the ICU and underlying factors currently lacks in the literature^22–24^, where sex differences in survival and the role of potentially confounding factors remain unknown^7,8^. Therefore, we investigated the association between sex and ICU survival in a Western European cohort of SARS-CoV-2 infected patients, adjusting for age, disease severity, obesity, smoking, comorbidities, and anti-infection/inflammatory therapy.

## Results

**Figure 1:**
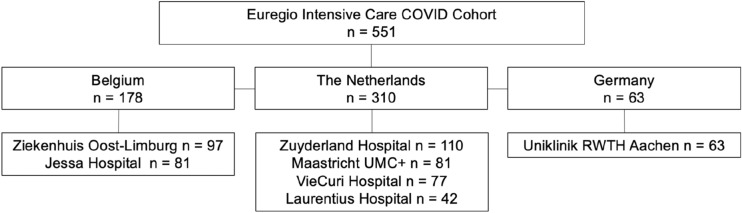
Flow chart.

From the 2nd of March 2020 to the 12th of August 2020, 551 patients with COVID-19 pneumonia were admitted to seven ICUs mentioned above in Western Europe (Fig. 1), 434 (79%) were mechanically ventilated. During illness, 18 (3%) patients were transferred within the Euregio and thus admitted to two or three of the Euregio ICUs (due to lack of bed availability and/or tertiary care referral for extracorporeal membrane oxygenation consideration). The number of females in the whole cohort was 159 (29%). The mean age was comparable between females and males (64.1 ± 12.6 vs 66.0 ± 10.5 years, p-value 0.095), as were the presence of dyslipidemia, diabetes mellitus, hypertension, chronic liver disease, chronic lung disease, and chronic renal disease (Table 1, p-values > 0.05). However, females were more often obese than males (42% vs 28%, p-value 0.001) and reported smoking less often than males (15% vs 22%, p-value 0.042). Acute Physiology And Chronic Health Evaluation II (APACHE II) scores did not differ between females and males (15.7 ± 5.2 vs 16.3 ± 5.6, p-value 0.305).

**Table 1:** ICU admission characteristics stratified for females and males of the full Euregio Intensive Care cohort.

	Females	Males	p-value
Number of patients	159	392	
Age, years	64.1 ± 12.6	66.0 ± 10.5	0.095
Height, m	1.63 ± 0.08	1.78 ± 0.08	< 0.001
Weight, kg	80.5 ± 17.7	90.0 ± 16.1	< 0.001
Body mass index, kg/m^2^	30.1 ± 6.4	28.6 ± 4.7	0.007
Obesity, n (%)	66 (42)	109 (28)	0.001
Dyslipidemia, n (%)	41 (26)	108 (28)	0.500
Diabetes Mellitus, n (%)	40 (25)	101 (26)	0.882
Hypertension, n (%)	76 (48)	184 (47)	0.805
Smoking, n (%)	24 (15)	88 (22)	0.042
Chronic liver disease, n (%)	1 (1)	3 (1)	1.000^a^
Chronic lung disease, n (%)	34 (21)	67 (17)	0.238
Chronic renal disease, n (%)	23 (15)	45 (12)	0.334
Patients admitted from the emergency department/hospital ward/by transport, n	54/77/28	130/200/62	0.816
Patients from Belgium/ the Netherlands/Germany, n	60/77/22	118/233/41	0.061
APACHE II score	15.7 ± 5.2	16.3 ± 5.6	0.305
Antibacterial therapy, n (%)	145 (91)	378 (96)	0.011
Antiviral medication, n (%)			0.527^a^
Oseltamivir, n (%)	6 (4)	8 (2)	
Lopinavir/ritonavir, n (%)	5 (3)	13 (3)	
(Hydroxy)chloroquine, n (%)	82 (52)	234 (60)	0.081
Remdesivir, n (%)	2 (1)	0 (0)	0.083^a^
Interleukin inhibitors, n (%)	6 (4)	15 (4)	0.972
Steroids, n (%)	56 (35)	116 (30)	0.223

Data are presented as mean ± SD, median [IQR], or percentages. P-values for differences between sex are tested by independent Student's T-Test, Mann–Whitney U test or Chi-Square, as appropriate unless otherwise specified: ^a^Fisher's exact test. ICU, Intensive Care Unit. The comprehensive data for the full cohort were complete, except missings for height (n = 27), weight (n = 33), BMI (n = 37), obesity (n = 20), dyslipidemia (n = 108), hypertension (n = 1), smoking (n = 96), antiviral medication (n = 3), interleukin inhibitors (n = 1), and steroids (n = 4).

**Figure 2:**
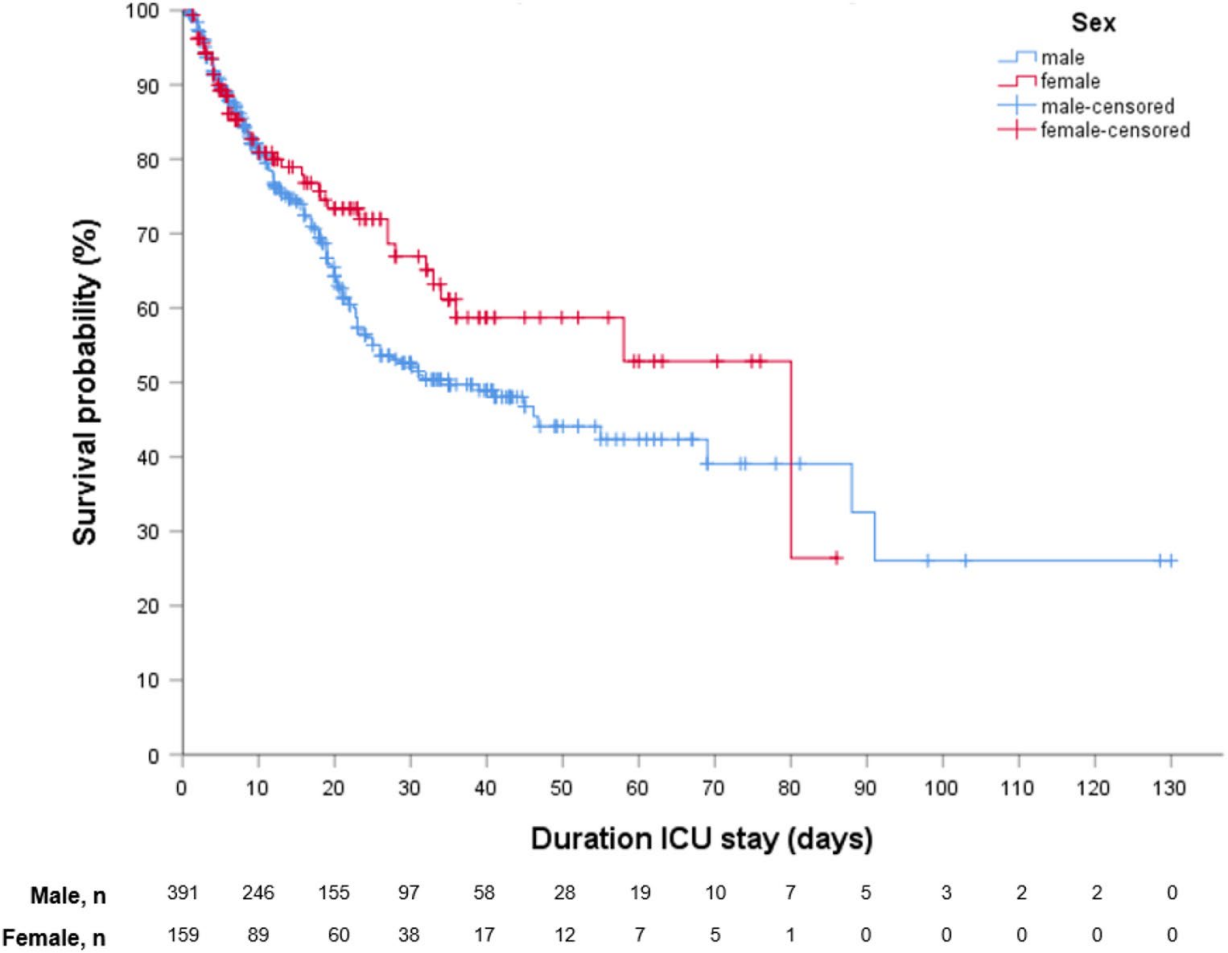
Kaplan–Meier survival estimate by sex ICU, Intensive Care Unit. The Kaplan–Meier survival curves show that more females survive the ICU than males, while the curves cross around 80 days with a very low number of events by then. Number at risk (n) = 550 as 1 patient missed data on duration of ICU stay.

ICU length of stay was comparable between females and males (12.4 [5.0–28.0] vs 16.0 [7.0–30.0] days, p-value 0.347). During ICU stay, females required less often invasive mechanical ventilation (72% vs 82% respectively, p-value  0.010) and antibacterial therapy than males (91% vs 96%, respectively, p-value 0.011). Administration of other anti-infection/inflammatory drugs did not differ between the sexes (Table 1). The ICU mortality rate was 12% lower in females than males (27% vs 39%, p-value < 0.008) (Table 2, Fig. 2). The Kaplan–Meier survival curves showed that more females survived in the ICU than males, while the curves crossed around 80 days of admission (although with a very low number of events) (Fig. 2).

**Table 2:** ICU outcomes of the Euregio Intensive Care cohort stratified for females and males.

	Females	Males	p-value
Number of patients	159	392	
ICU death, n (%)	43 (27)	153 (39)	0.008
Length of ICU stay, days	12.4 [5.0–28.0]	16.0 [7.0–30.0]	0.347
Invasive mechanical ventilation, n (%)	114 (72)	320 (82)	0.010
Length of invasive mechanical ventilation, days	15.8 [8.20–27.0]	16.5 [8.7–27.9]	0.477

Data are presented as median [IQR], or percentages. P-values for differences between sex are tested by the Mann–Whitney U test or Chi-Square, as appropriate. ICU, Intensive Care Unit. The comprehensive data for the full cohort were complete, except missings for length of ICU stay (n = 1) and length of invasive mechanical ventilation (n = 4).

In a mixed-effects crude logistic regression model with a random center effect, females had a lower odds ratio for mortality than males (OR 0.59 (95%CI 0.39–0.89), Table 3, model 1), suggesting higher ICU survival rates in females than males. Adjustment for age and APACHE II score (OR 0.62 (95%CI 0.39–0.96), model 2), and additional adjustment for the presence of obesity, dyslipidemia, diabetes mellitus, hypertension, smoking, chronic liver disease, chronic lung disease, and chronic renal disease (OR 0.63 (95%CI 0.40–0.99), model 3), or antibacterial therapy, antiviral medication, (hydroxy)chloroquine, remdesivir, interleukin inhibitors, and steroids (OR 0.63 (95%CI 0.39–0.99), model 4) did not change the results. Similar results were observed in the subgroup of mechanically ventilated patients (n = 434) (Table 3). Between-center variances in model intercepts were not statistically significantly different (p-values for models 2, 3 and 4 ≥ 0.17), which means that baseline characteristics of sexes did not explain the observed sex differences in mortality. When adding interaction terms for sex and country and sex and obesity to the models, no effect modifications were observed (p-values for interaction > 0.23 and 0.84, respectively). A sensitivity analysis with missing data handled by multiple imputation showed similar results.

**Table 3:** The association between sex and ICU death by mixed-logistic regression analyses.

	Full cohort n = 551			Mechanically ventilated subcohort n = 434		
	OR	95% CI	p-value	OR	95% CI	p-value
Model 1. The crude model with a random intercept for hospital	0.59	0.39–0.89	0.012	0.56	0.36–0.89	0.014
Model 2. Model 1 + age and APACHE II score	0.62	0.39–0.96	0.032	0.57	0.35–0.92	0.023
Model 3. Model 2 + obesity, dyslipidemia, diabetes mellitus, hypertension, smoking, chronic liver disease, chronic lung disease and chronic renal disease	0.63	0.40–0.99	0.044	0.61	0.37–1.01	0.052
Model 4. Model 2 + antibacterial therapy, antiviral medication, (hydroxy)chloroquine, remdesivir, interleukin inhibitors, and steroids	0.63	0.39–0.99	0.047	0.58	0.35–0.96	0.036

Data are odds ratios (OR) with 95% confidence intervals (95% CI) for females compared to males (as reference). A lower OR indicates an increased survival rate for females. Missings (for the full cohort: obesity (n = 20), dyslipidemia (n = 108), hypertension (n = 1), smoking (n = 96), antiviral medication (n = 3), interleukin inhibitors (n = 1), and steroids (n = 4)) were included in model 3 and 4 as separate category.

In addition, sensitivity analyses of excluding transported patients displayed similar effect estimates, although statistical power was somewhat reduced (Supplementary Table S1).

## Discussion

Although patients are individuals, traditionally, we tend to categorize them according to their disease or condition and treat them in the same "diagnosis category" using one-size-fits-all interventions. However, how a disease may unfold in an individual patient has many dimensions, and heterogeneity has major implications on disease course and outcome^25^. For COVID-19, heterogeneity in the course of the disease and complications has been observed^9,26^, ranging from typical flu-like symptoms to critical illness requiring intensive care admission and death^27,28^.

In this cohort study of 551 COVID-19 patients admitted to the ICUs of seven hospitals in three Western European countries, we demonstrated that females had a 40% greater chance to survive ICU stay than males, independent of age, the severity of acute critical illness, obesity, smoking, major comorbidities, administered anti-infection/inflammatory therapy, and country. The results were similar for the subgroup of invasively mechanically ventilated patients. No effect modifications for sex with country and sex with obesity were present. Secondly, we observed that females were the minority in the Euregio cohort, representing 29% of the study population.

The prevalence of females in our study is in line with the reported prevalence in previous COVID-19 studies in ICU patients^7^. In a multicenter observational study in Italian ICU patients^8^, 20.1% (95%CI 18.9–21.3) of the study population was female with a median age of 64 years, which is in line with the age in our study. However, the reported mortality of approximately 50% is higher than in our study, which may relate to the higher prevalence of comorbidities in their cohort. Nevertheless, in agreement with our findings, they found that the male sex was significantly associated with mortality (HR 1.57 95%CI 1.31–1.88), although they did not adjust for the severity of disease.

Overall, evidence points towards a slightly lower prevalence of symptomatic COVID-19 in females than males (45% vs 55%, respectively)^29^, with males having a consistently three times higher odds for ICU admission and once admitted an up to 40% higher mortality rate compared with females, both in line with our findings^6^. Notably, these findings are not unique for COVID-19. In fact, higher mortality rates for males were also observed in previous Middle East Respiratory Syndrome Coronavirus and Severe Acute Respiratory Syndrome Coronavirus 1 outbreaks^30,31^. Several studies have demonstrated that elderly patients with comorbidities are at increased risk of dying from COVID-19^32–34^. Additionally, underlying health conditions such as cardiovascular disease, hypertension, diabetes mellitus, smoking, and obesity were associated with an increased risk of mortality^35–37^. Our study shows a higher odds of mortality for males, as other studies do, but is distinctive since results are independent of age, smoking, obesity, comorbidities, APACHE II scores (i.e., the classification system used to assess disease severity)^38^, anti-infection/inflammatory therapy, and country, indicating that sex might be associated with ICU outcome independently of disease severity at ICU admission.

Although COVID-19 seems to be linked to multiple "traditional" risk factors, its presentation is multidimensional, with sex and gender being essential determining factors. Although it had been suggested that the sex disparities in COVID-19 were due to higher smoking and comorbidity rates observed in males^39^, we show that the association between sex and ICU outcome is independent of disease severity, “traditional risk factors,” and treatments. As treatment could be considered as a mediator, instead of a confounder, in the reported models, the results suggest that treatments evaluated are not a clinically and statistically significant contributor to the causal pathway between sex and ICU outcome. Even more striking is the higher prevalence of obesity seen in females in our cohort, which has been associated with worse outcomes in earlier studies, while it did not affect the better survival of females compared to males in our study.

These findings support the theory that the pathophysiology of SARS-COV-2 infection may differ between both sexes in the ICU setting^9,16^. Indeed, evidence on several sex-specific interacting mechanisms on immunological, hormonal, and cardiovascular pathophysiology is accumulating^4,14,29,40–50^. Since many genes involved in the immunological response to infection are present on the X chromosome, the XX and XY genetic constitutions could also potentially contribute to COVID-19 severity^40,48^. Females show a more rapid and aggressive immune response to pathogens with a lower degree of systemic inflammation, which facilitates viral clearance^41,51^. Moreover, females have a more robust T-cell activation during SARS-CoV-2 infection, whereas males have higher plasma levels of innate immune cytokines and increased non-classical monocyte cell populations induction. Both observations are correlated with increased severity in males^49,50^. In addition, female and male steroid and sex hormones could play a contributory role in pathogenesis. Estrogen in females, for instance, can have immune-enhancing effects, while testosterone in males can exert immune-suppressive effects. Furthermore, the expression of receptors that determine viral cell entry, such as transmembrane protease serine 2 (TMPRSS2) and angiotensin-converting enzyme (ACE), is affected by sex hormones and lower in females than in males^40–44^. TMPRSS2 is enhanced by testosterone and may play a role in delayed viral clearance^41,42^, whereas the ACE2 receptor, exerting protective effects in the heart, lungs, kidneys, and guts by deactivating the effects of the renin-angiotensin system (RAS)^51^, is encoded on the X chromosome and downregulated by estrogens. Thus, females show a reduced propensity to upregulate RAS activity in COVID-19, which could contribute to higher disease severity in males, as RAS overactivation contributes to pathogenesis in cardiovascular disease and potentially COVID-19^51^.

Our study shows for the first time that, *independent* of cardiovascular comorbidities, females have a survival benefit for COVID-19 once admitted to the ICU. Sex differences in the prevalence of subclinical and yet undiagnosed underlying cardiovascular comorbidities may still play a role, as we cannot entirely exclude residual confounding^21^, while our adjustments were comprehensive. However, it is unlikely that residual confounding thoroughly explains the observed sex difference.

### Strengths and limitations

Several studies have shown that females have higher survival rates in heterogenic population-based data sets^4^. Our study, however, is the first to demonstrate that a higher survival rate of females is maintained after admission to a Western European ICU, and more importantly, independent of age, disease severity, obesity, smoking, comorbidities, anti-infection/inflammatory therapy, and country. Sensitivity analyses, excluding transported patients to and out of Euregio, showed similar results, which reduces bias due to loss of ICU follow-up beyond Euregio. In addition, the identification of transports within Euregio reduces bias due to including the same patient twice in the models. Although we collected variables from healthcare systems, our data collection was complete (models 1 and 2), except for a few missings for some potential confounders (obesity and hypertension), while smoking and dyslipidemia only were less complete (> 5% missings) (models 3). Therefore, we additionally used multiple imputation to handle missing data appropriately^52–55^. The data collection process met high standards, and collected healthcare data were of high quality using a predefined protocol addressing the present hypothesis^56^. Moreover, we used multivariable adjustments to address a comprehensive set of potential confounders. Nevertheless, the study cannot rule out that residual confounding has biased the reported associations^21,57,58^. We did not follow-up on patients over a predefined time period and classified survivors as those who were discharged from the ICU or transported.

As patients may have died after ICU discharge or transport, we feel that a survival analysis including time cannot be appropriately performed and would present invalid results, which is a limitation of the study. Consequently, no conclusion can be drawn on COVID-19 progression in the ICU based on our data. We recognize that our findings are limited to the ICU population of the Euregio, thereby limiting generalization to other contexts outside the ICU, such as other patient populations (e.g., from national registries or hospitalized) and regions^59,60^. Admission to the ICU is the result of a selection process, which depends on many factors, including those potentially associated with sex and possibly causes index-event bias^61,62^. If so, the observed association between sex and ICU survival could function in this selection process. Thus, as an alternative to the pathophysiological explanation for the observed sex differences in ICU outcome as discussed above, the results could also be explained by doctors’ decisions regarding admission of patients to the ICU. Unfortunately, our dataset did not include information on the source population (i.e., all patients admitted for COVID-19 to the seven hospitals), and we thus cannot investigate whether selection has occurred. In addition, the results cannot be generalized beyond the ICU. As the large majority of inhabitants of Euregio are of Caucasian descent, diversity due to ethnicity within our cohort was too low for subanalysis. This variable was not considered for the collection, while it was not registered in a standardized way within each of the hospitals. Finally, the observational study design limits to conclude with regard to causality and the relation with subclinical yet undiagnosed underlying cardiovascular disease.

In this study in the Euregio, we demonstrate that females, once admitted to the ICU with COVID-19 pneumonia, have a 40% higher survival rate relative to males and that this association is independent of age, disease severity, obesity, smoking, comorbidities, anti-infection/inflammatory therapy, and country of residence. Understanding the relation between sex and COVID-19 implies recognizing diversity in the role of both biological and social factors in the risk of infection and disease, clinical presentation, the severity of outcomes, and patient selection at the individual as well as population levels. A sex-specific prediction, prognostic and therapeutic approach and sex-stratified analyses strategies should be implemented in future ICU studies, while further studies on sex-specific mechanistic pathways in SARS-CoV-2 are warranted.

## Methods

The Euregio Intensive Care COVID cohort, part of the Euregio Covid Data Platform (CoDaP) project, was initiated in early March, at the beginning of the first wave of the COVID-19 pandemic. Seven neighboring ICUs (Supplementary Table S2) in Belgium, the Netherlands, and Germany collaborated and planned to share their data in a predesigned way^63^.

We consecutively included patients with COVID-19 pneumonia and respiratory failure admitted to the ICU of any of the seven hospitals between the 2nd of March 2020 and the 12th of August 2020 (Fig. 1). At the time of admission, all patients presented with signs and symptoms of viral pneumonia. The diagnosis was confirmed by a positive PCR for SARS-CoV-2 and/or (for the Netherlands only) a positive score on chest CT scan of 4–5 based on the COVID-19 Reporting and Data System (CO-RADS) score as confirmed by a radiologist^64^. Patient admission occurred via the emergency department, non-ICU wards, or from other (international) ICUs, in the latter case either for tertiary care referral or due to lack of bed availability. Follow-up ended when patients were either discharged from ICU or died, from the 11th of March to the 2nd of September.

### Data collection

At the beginning of the pandemic, the number of variables was determined in the focus of interest. These included baseline demographic and clinical characteristics, laboratory values, interventions, and outcome variables, which were predefined and routinely obtained during patients' stay in the above-mentioned ICUs. The main outcome variable for the present study was ICU death. Variables were collected mostly retrospectively and pseudo-anonymized at the collecting hospital. Pseudo-anonymization of data is a widely accepted method that aims to secure the patient's privacy about his/her healthcare data while ensuring the possibility to re-collect data at the dispatching hospital. Subsequently, data were shared with Maastricht UMC+, the coordinating center within CoDaP, using electronically secured data transfer methods and stored on a secured hospital hard drive. Data access was only permitted for the primary investigators.

Data cleaning started by checking whether each variable of the study protocol was present in each of the seven datasets. Next, each hospital dataset was standardized (i.e., standardization of the variable names, characteristics, and units). When a variable was missing or inconsistencies were encountered, contact with investigators of the dispatching hospital led to re-collecting, re-calculation, and re-sending of those variables^65^. The seven datasets were then merged into one dataset. Patients transferred between the ICUs of the seven Euregio hospitals were identified. Their whole ICU stay was attributed to the primary admitting ICU to prevent duplicate patient data and missing data by combining baseline and outcome data. Finally, each of the pre-final Euregio cohort variables dataset was evaluated for outliers through running queries, which was checked with the source dataset of the dispatching hospital and corrected if possible and appropriate or defined as missing. Obesity was defined as a body mass index (BMI) equal to or larger than 30 kg/m^2^. Smoking was defined as either active smoking or a history of smoking. Comorbidities were defined either as a history of a medical diagnosis or the actual use of medication for such medical diagnosis before ICU admission.

### Statistical analyses

IBM SPSS Statistics version 25 (IBM corporation, NY, USA) was used for all analyses. The full cohort was categorized into females and males, and data were presented. The number of missings per variable is reported in the table legends. Descriptive statistics were performed on available data only. Differences between females and males were analyzed using the independent Student's T-test, Mann–Whitney U test, Chi-Square, or Fisher's exact test, as appropriate. With a random intercept for a center, mixed-effects logistic regression was used to investigate the association between sex and ICU survival. Crude models were first adjusted for age and APACHE II score, then for obesity, dyslipidemia, diabetes mellitus, hypertension, smoking, chronic liver disease, chronic lung disease, and chronic renal disease^21^, and eventually for antibacterial therapy, antiviral medication such as oseltamivir, ritonavir/lopinavir, (hydroxy)chloroquine and remdesivir, interleukin inhibitors, and steroids. Confounders were treated as continuous or categorical variables, and missing data were included as a separate category. As this method could lead to bias in observational studies^52–55^, a sensitivity analysis was performed by handling missing data by multiple imputation. Missing values in the variables included in the model were explored, and the imputation number was based on the percentage of patients with at least one missing value. Predictive mean matching was the method of choice, and variables with missing data, auxiliary variables, and outcome variables were added to the model. Effect modifications by sex with country and sex with obesity were investigated by adding interaction terms for categories of sex and country and sex and obesity to the models. Analyses were performed for the full Euregio cohort and the mechanically ventilated patients of the cohort. Subsequently, sensitivity analyses were performed by excluding patients who were transported from a hospital outside the Euregio to our participating Euregio hospitals (i.e., due to missing baseline data), and by additionally excluding patients who were transported out of the Euregio (i.e., due to missing ICU follow-up data from hospitals beyond Euregio). Finally, for illustration, Kaplan–Meier survival curves were created. A two-sided p-value of < 0.05 and a p-interaction of < 0.1 were considered statistically significant.

### Ethical approval

Ethical approval was obtained from the medical ethics committee (METC 2020–1565/3 00 523) of Maastricht UMC+. The study was performed in accordance with the General Data Protection Regulation (GDPR) act and the national data privacy laws. Patient data were collected according to good clinical practice and in accordance with relevant guidelines and regulations. Informed consent was waived by the METC of Maastricht UMC+. However, each of the participating hospitals had its own policy and approach. For example, in Maastricht UMC+, the board of directors adopted a policy to inform patients and ask their consent to use collected data^63^. Data sharing agreements between Maastricht UMC+ and each hospital were drawn up by legal officers of Maastricht UMC+ and Clinical Trial Center Maastricht (CTCM) and subsequently tailored to each hospital. Investigators, heads of ICU departments, and the hospital board of directors of the Maastricht UMC+ and the other hospitals signed the final data-sharing agreement.

## Supplementary Information

Supplementary Information.

## Data Availability

The datasets generated and/or analyzed during the current study are not publicly available due to data sharing agreements of the participating hospitals. Individual patient data and the pseudo-anonymized dataset will not be made available to others. Only data for the full cohort or a particular subcohort will be published and shared after the provision of a research proposal and signed data access agreement of each participating hospital.
