# Supplementary material for: Better COVID-19 Intensive Care Unit survival in females, independent of age, disease severity, comorbidities, and treatment
Source: Sci Rep. 2022 Jan 14;12:734. doi: 10.1038/s41598-021-04531-x (PMC8760268; doi:10.1038/s41598-021-04531-x)
Supplement: Supplementary file 1 — Supplementary Information. [file 41598_2021_4531_MOESM1_ESM.docx]

**Better COVID-19 Intensive Care Unit survival in females, independent of age, disease severity, comorbidities, and treatment**

Daniek A.M. Meijs^1,2^*, Bas C.T. van Bussel^1,3^, Björn Stessel^4,14^, Jannet Mehagnoul-Schipper^5^, Anisa Hana^2^, Clarissa I.E. Scheeren^6^, Sanne A.E. Peters^7-9^, Walther N.K.A. van Mook^1,10,11^, Iwan C.C. van der Horst^1,11^, Gernot Marx^12^, Dieter Mesotten^13,14^, Chahinda Ghossein-Doha^1,11,15,16^, CoDaP investigators^1-17^

Affiliations:

1. Department of Intensive Care, Maastricht University Medical Center (Maastricht UMC+), Maastricht, the Netherlands
2. Department of Intensive Care, Laurentius Ziekenhuis, Roermond, the Netherlands
3. Care and Public Health Research Institute (CAPHRI), Maastricht University, the Netherlands
4. Department of Intensive Care, Jessa Hospital, Hasselt, Belgium
5. Department of Intensive Care, VieCuri Medisch Centrum, Venlo, the Netherlands
6. Department of Intensive Care, Zuyderland Medisch Centrum, Heerlen/Sittard, the Netherlands
7. Julius Center for Health Sciences and Primary Care, University Medical Center Utrecht, Utrecht, the Netherlands
8. The George Institute for Global Health, Imperial College London, London, United Kingdom
9. The George Institute for Global Health, University of New South Wales, Sydney, Australia
10. Maastricht UMC+ Academy for Postgraduate Medical Education, Maastricht, the Netherlands
11. Cardiovascular Research Institute Maastricht (CARIM), Maastricht, the Netherlands
12. Department of Intensive Care, University Hospital RWTH Aachen, Aachen, Germany
13. Department of Intensive Care, Ziekenhuis Oost-Limburg, Genk, Belgium
14. UHasselt, Faculty of Medicine and Life Sciences, Diepenbeek, Belgium
15. Department of Cardiology, Maastricht UMC+, Maastricht, the Netherlands
16. School for Oncology and Developmental Biology, Maastricht UMC+, Maastricht, the Netherlands
17. Department of Clinical Epidemiology and Medical Technology Assessment, Maastricht UMC+, Maastricht, the Netherlands

**Supplementary Table S1. The association between sex and ICU death by mixed-logistic regression analyses, excluding patients transported to the Euregio and excluding patients transported to and out of Euregio**

|  | Full cohort excluding patients transported to Euregio  n = 461 | | | Full cohort excluding patients transported to and out of Euregio  n = 448 | | |  |
| --- | --- | --- | --- | --- | --- | --- | --- |
|  | OR | 95% CI | p-value | OR | 95% CI | p-value |  |
| Model 1. The crude model with a random intercept for hospital | 0.66 | 0.42-1.04 | 0.074 | 0.64 | 0.41-1.01 | 0.054 |  |
| Model 2. 1 + age and APACHE II score | 0.69 | 0.42-1.12 | 0.133 | 0.65 | 0.40-1.07 | 0.087 |  |
| Model 3. 2 + obesity, dyslipidemia, diabetes mellitus, hypertension, smoking, chronic liver disease, chronic lung disease, and chronic renal disease | 0.70 | 0.42-1.16 | 0.166 | 0.65 | 0.39-1.09 | 0.103 |  |
| Model 4. 2 + antibacterial therapy, antiviral medication, (hydroxy)chloroquine, remdesivir, interleukin inhibitors, and steroids | 0.70 | 0.42-1.12 | 1.17 | 0.67 | 0.40-1.11 | 0.120 |  |
| Data are odds ratios (OR) with 95% confidence intervals (95%CI) for females compared to males (as reference). A lower OR indicates an increased survival rate for females. | | | | | | | |

**Supplementary Table S2. Overview ICUs participating in the CoDaP project**

| Belgium | The Netherlands | Germany |
| --- | --- | --- |
| Ziekenhuis Oost-Limburg (Genk) | Zuyderland Hospital (Heerlen/Sittard) | University Hospital RWTH Aachen |
| Jessa Hospital (Hasselt) | VieCuri Hospital (Venlo) |  |
|  | Laurentius Hospital (Roermond) |  |
|  | Maastricht University Medical Center+ (Maastricht UMC+, (Maastricht) |  |

ICUs, Intensive Care Units; CoDaP, Covid Data Platform; RWTH, Rheinisch-Westfälische Technische Hochschule.
